# Using Sequence-Approximation Optimization and Radial-Basis-Function Network for Brake-Pedal Multi-Target Warping and Cooling

**DOI:** 10.3390/polym14132578

**Published:** 2022-06-25

**Authors:** Hanjui Chang, Guangyi Zhang, Yue Sun, Shuzhou Lu

**Affiliations:** 1 Department of Mechanical Engineering, College of Engineering, Shantou University, Shantou 515063, China; 20gyzhang1@stu.edu.cn (G.Z.); suny09222022@163.com (Y.S.); 21szlu@stu.edu.cn (S.L.); 2 Intelligent Manufacturing Key Laboratory of Ministry of Education, Shantou University, Shantou 515063, China

**Keywords:** warpage, cycle time, sequential approximate optimization, conformal cooling channel, Pareto boundary, brake pedal

## Abstract

This paper uses a multi-objective optimization method to optimize the injection-molding defects of automotive pedals. Compared with the traditional automotive pedal material, aluminum alloy, the polymer pedal containing glass fibers not only reduces the aluminum pedal by at least half, but also improves the strength and hardness of the fibers by adjusting the orientation of the fibers in all directions. Injection factors include: filling time, filling pressure, melt temperature, cooling time, injection time, etc. For the optimization process influencing factors, herein, we focus on warpage analyzed via flow simulation, and setting warpage parameters and cycle time as discussed by setting different cooling distributions, pressures and temperature schemes. The multi-objective optimization design was mainly used to describe the relationship between cycle time and warpage, and the Pareto boundary was used for cycle time and warpage to identify the deviation function and radial-basis-function network. We worked with a small DOE for building the surface to run SAO programming—which improved the accuracy of the response surface by adding sampling points—terminating the time when the warpage value met the solution requirements, to find out the global optimal solution of the warpage value under different cooling times. Finally, the results highlighted four influencing parameters that match the experimental image of the actual production.

## 1. Introduction

The molding conditions of the injection pedal can also affect the productivity, cycle time and energy consumption of the molding process. The molding conditions of the injection pedal are closely related to other factors that determine the quality of plastic products, such as material, parts design, mold and other factors. The injection-molding factors studied in this experiment include: filling time, packing pressure, melt temperature, cooling time, injection time, etc.

The quality of a molded part depends on the process parameters and the properties of the plastic material. Finding the best process parameters can effectively reduce the number of cycles and improve product quality. The determination of process parameters in the actual production largely depends on the experience of the plastic engineers and the actual site commissioning. This method does not effectively ensure the accuracy of the process parameter values. With plastic it is difficult to predict thermal bonding properties, how to set the optimal molding conditions, and how to maximize the required product quality, which are crucial. Therefore, the process parameters are usually selected from the manual and then adjusted using the trial-and-error method. As can be seen, trial-and-error is expensive and time consuming. For manual lysis methods, many mathematical equations have been developed to derive the inherent process parameters for injection molding. However, when analytical equations involve many simplifications, they often do not meet the reliability requirement due to the complexity of the injection process. As a result, many researchers are trying to find ways to optimize the parameters of the molding process.

The production advantages of plastic products are light weight, high stiffness and high productivity. Therefore, compared with the commonly used all-aluminum pedal, the brake pedal in polymer materials will have the advantage of lighter weight and better strength. The three main stages of the widely used plastic injection-molding (PIM) [1] process are: the filling stage, pressure holding stage and cooling stage. First, during the filling stage, the molten plastic is filled into the cavity type of the mold under the pressure of the injection-molding machine. Secondly, in the holding stage, the molten plastic is compressed at very high pressure to obtain the shape of the part. The molten plastic solidifies during the cooling phase. The final cooling of the formed solid plastic is lifted out of the mechanism. Therefore, in order to improve the cycle time and warping time simultaneously, it is necessary to optimize the melt temperature, pressure-retaining pressure, pressure fixation time, injection time, cooling time and other process parameters. In addition, deformation, volume shrinkage and welding line are the main defects that should be avoided for size accuracy and the appearance of the finished product, in order to obtain higher-quality products. The design function of the pedal is mainly to optimize the design of the cooling pipe and shorten the entire product-molding cycle, including filling, pressure, cooling, etc. Deformation and cooling shrinkage also need to be reduced, which can be further optimized by optimizing the design of the cooling pipe for better injection-molding parameters and product quality. In the design process of the conformal cooling waterway, it is necessary to continuously adjust the relevant parameters, such as the diameter of the cooling-water pipe, the number of cooling-water pipes, the arrangement of the cooling-water circuit, etc.; constantly adjust the parameters in combination with the warpage and cooling results of the simulation experiment; and finally, obtain the optimal cooling circuit design. This study optimizes the automotive automatic brake-pedal injection-molding parameters and compares the original parameters with the optimized parameter results; discusses the influence of these parameters on the product warpage shrinkage and molding cycle; and sets up different cooling channels, pressures and plastic temperature schemes.

## 2. Literature Review

In terms of reducing warpage deformation and obtaining optimal injection-molding parameters, this study requires multi-objective optimization, which is compared with other classical mathematical schemes in the following literature review process.

In 2004, Mohd Sapuan Salit [1] analyzed and calculated the possible configurations and geometries of brake pedals. Based on the performance of existing and suitable polymer-matrix composites, the results show that polyamide with short glass fibers can be the most suitable material for polymer-based composite brake pedals. By properly designing the polymer-matrix brake pedals, one can reduce the weight by 73%. In 2006, Hassan Kurtaran and Tuncay Erzurumlu [2] used a combination of process parameters organized by experimental design, which were analyzed via statistical full three-level analysis. According to the FEM results based on the analysis of variance (ANOVA) method, the most important process parameter affecting warping was the within-mode pressure value. The response-surface model (RSM) with an effective genetic algorithm yielded the best process parameter value, with an optimization rate of 38%. In 2007, Y. C. Lam [3] proposed a strategy to optimize injection-molding conditions using a mixture of gradient methods and genetic algorithms, which first derived elite solutions from the genetic algorithm optimization results to identify candidate local minimum regions. These solutions were searched as gradient methods for local minima from which the best solution was selected. The hybrid algorithm had more stable optimization results than single inheritance. In 2007, Shen Changyu [4] proposed a method that combined artificial a neural network with a genetic algorithm to improve the quality index of part volume shrinkage change, established an neural network (NN) model of volume shrinkage change and process conditions of 5-9-1 structural injection molding, and verified the optimization results of genetic algorithm through numerical experiments. In 2008, Yuehua Gao [5] ordered SAO based on the Kriging model to minimize the warping of a phone case. The rectangular grid of the spatial filling sampling strategy of the Krieger model was modified using the functional relationship established by the Kriging model between warping and different parameters. The results show that the optimization method can effectively reduce warping.

In general, population-based optimization techniques require a large number of function evaluations to find the global minimum and a set of Pareto-optimal solutions. This makes it difficult to apply these optimization techniques directly to actual design optimization issues in some cases due to time-consumption issues. Because classical mathematical planning requires the sensitivity of goals and constraints, it is not suitable for non-micro-problems. In addition, a functional evaluation of sensitivity and step determination needs to be calculated. Today, the time available for developing new products is decreasing, so it is best to reduce the computational time required for optimization.

In 2007, G. Gary Wang and S. Shan [6] summarized engineering design optimization using DOEs to set sample points in the design variable space, and evaluated the goals and constraints of these sample points via LHD. The response surface was constructed using a kriging and radial-basis-function (RBF) network, and finally the approximate optimal value was obtained by optimizing the response surface; this value was used as the approximate optimization result. In 2009, Y. Zhang Y.M. Deng [7] proposed a method to reduce the warpage defects of injection parts. Mode-pursuing sampling (MPS) to warp optimization allowed more sample points to be systematically generated within the current optimal solution neighborhood while statistically covering the entire search space. The design variables for molding time, melt temperature and mold temperature showed that warpage deformation can be effectively reduced and the calculation results were significantly reduced. In 2009, in a study by Chuang Li and Fu-Li Wang [8], using a step-by-step optimization method based on an RBF proxy model, an advanced expected improvement criterion was proposed to improve the overall performance of the optimization method. Packaging contour optimization was applied to the injection-molding process to achieve shrinkage uniformity of molded parts. The improved approach enables stronger global optimization performance and more accurate optimal solutions without the need for appropriate padding data. In 2010, Yuehua Gao [9] proposed an adaptive optimization method based on the Kriging proxy model, and the adaptive process was implemented via the Expected Improvement (EI) fill sampling standard. The guide allows the use of DOEs for global searches in a very short period of time, and to find best design. The results showed that the adaptive multi-objective optimization method can effectively reduce the warpage of the mobile phone case. In 2011, Satoshi Kitayama [10] and others proposed a sequence-approximation optimization (SAO) algorithm based on the RBF network. A new adaptive scaling technology was developed; a new density function was constructed using the RBF network to determine the sparse region between the sample points; the global minimum of the density function was used as the new sample point; and finally, the approximate global optimal solution was obtained. In 2012, Kurtalan [11] obtained a local minimum value by scaling the method if the optimal value of the response face continued to increase. Global and local approximations are improved via sequential sampling strategies, and improved algorithms can be used to find sparse regions. In the EI algorithm, regions with high uncertainty correspond to sparse regions. In order to find the sparse regions, it is necessary to adjust the parameters in the Gaussian function. In 2013, Chul Woo Park [12] performed injection-molding analysis of truck pedals based on the location of the gate. The runners were 5 mm and 100 mm high. There were four schemes: molding started from the round part of the brake-pedal shell, the brake shell with the straight part on both sides, the back of the brake case, and the part where the brake case was mounted on the body. Result had a minimum pressure of 30 Mpa.

It is important to use global optimization techniques to find high-precision global minima, which often require a lot of function calculations. However, if the objectives and constraints are ambiguous, but can be evaluated via computationally intensive numerical simulations, the response surface (called metamodeling) is an attractive method for finding approximate global minima through a small amount of functional evaluation. Warping deformation is a nonlinear implicit function of process conditions, usually solved using finite-component (FE) equations, and the workload is very heavy. The Kriging model establishes an approximate function relationship between warping and process conditions, replacing the expensive warping reanalysis during optimization. The SAO method based on radial-basis-function network can meet the optimization requirements of injection-molding process parameters.

In 2014, Mohd Nizam Sudin [13] used CATIA V5 solid modeling software to generate a digital model of an existing brake pedal. Topology optimization under linear statical analysis was carried out using Altair OptiStruct software. A new lightweight brake-pedal design scheme was proposed. The results of the study showed that the newly designed brake pedal weighed 22% less than the existing brake pedal without sacrificing performance requirements. In 2014, Satoshi Kitayama [14] employed the SAO method using radial-basis-function networks. Taking the mold temperature and melt temperature as the design variables, short lenses of molten plastic that were not filled into the cavity were considered design constraints. The temperature distribution was analyzed, which showed that the variable pressure distribution could reduce the plastic temperature difference of the melt and effectively reduce warpage deformation. In 2014, Zhang [15] applied the kriging method to warpage and seal optimization through the pressure curve of shrinkage uniformity. They used a radial underlying function network to build the response surface. Additionally, in order to find undeveloped areas, they used the expected improvement function. The optimization rate of warpage results reached 28%. In 2014, Jian Zhao [16] proposed a two-stage multi-objective optimization framework based on Pareto-optimized injection-molding process parameters. The first phase used an IEGO algorithm to approximate the nonlinear relationship between machining parameters and part-quality measurements. In the second phase, NSGA-II based on non-dominant sequencing was used to look for better convergence to the boundary of true Pareto. The double Pareto boundary indicated a significant relationship between warpage and volumetric shrinkage. In 2014, Wen-Chin Chen and Deni Kurniawan [17] used the Tamakou method, reverse propagation neural network (RPNN), genetic algorithm (GA), and particle group optimization in combination with a genetic algorithm (PSO-GA) to find the best parameter settings, and in the first phase of optimization, signal-to-noise ratio predictors and genetic algorithms were used to reduce the variance of mass characteristics. In the second stage of optimization, an S/N ratio predictor and mixed PSO-GA quality predictor were used to find the best parameter settings for process quality characteristics and stability, and the proposed system not only improved the quality of plastic parts, but also effectively reduced the variability of the process.

It is important to note that the Tiankou method can find the best combination of process parameters, but the best process parameters cannot be found. Chen and Cunhawan recommend two-stage optimization using the Tiankou method and PSO-GA, wherein the Tiankou method is used to select the process parameters for the first stage, while the PSO-GA is used to determine the parameters for the second phase of the optimal process. Therefore, in order to obtain a high-precision response surface, it is often necessary to employ a large-scale experimental design. In recent years, the sequence-approximation optimization method has been widely used. In SAO, a small DOE is used first to build a response surface. One can then find the best value for the response face. The SAO algorithm is terminated if the terminal criteria set by the designer are met. Note that the number of sampling points is equivalent to the number of simulated runs. In addition, some new sampling points are added to improve the accuracy of the response surface. Through the iterative process described above, a highly accurate global optimal solution can be found. Therefore, the orderly SAO of repeatedly building and optimizing response surfaces has become a common method for determining the best process parameters. In SAO, the response surface is repeatedly built and optimized by adding several new sampling points. One can then find a highly accurate global minimum. Doing this using response surfaces and neural networks is a “one-step” optimization set that does not require iteration. Build the response surface repeatedly by adding new sampling points until the final criteria set by the decision maker are met. Compared to the classic response-surface method described above, one can achieve high accuracy approximate to the lowest global value by adding new sampling points. However, when using SAO to obtain high-precision data, it is often necessary to first consider the first target, which is to add the best point of the response face as the new sampling point. This results in high-precision local approximations. Adding new sampling points to sparse areas produces global approximations. This addition prevents one from falling into the local minimum. SAO is used to identify Pareto boundaries with a small number of simulations.

In general, the accompanying cooling channel improves cooling performance, cycle times and distortion. However, we do not know how to design subsequent cooling channels. In other words, the design of the accompanying cooling channels depends, to a large extent, on the designer’s experience. In addition, redesigning cooling channels is expensive and difficult to try. Therefore, it is necessary to use single-target or multi-target optimization to obtain an optimally shaped cooling waterway. The first CAE simulation can display the performance output of conformal cooling channel (CCC) according to different targets such as cooling time, warping thickness, temperature distribution and thermal stress. By evaluating the resulting output, one can obtain more information about the impact of different CCC designs, but the simulation of the application may not be sufficient to obtain the best design conditions. Optimization is the step to determine the optimal design conditions for CCC.

In 2010, Hong-Seok Park [18] proposed a conformal cooling channel with a bezel array, and a system of approximate equations was developed to represent the relationship between the cooling channel configuration, mold material, mold thickness, and temperature distribution within a particular polymer. The appropriate physical mathematical model was established through experimental design, and the optimization process of obtaining the target mold temperature and uniform temperature distribution and minimizing cooling time was significantly improved. In 2014, Choi [19] implemented an advanced optimization method developed using a CCC, in which minimizing the average mold temperature of the cycle and obtaining temperature uniformity were the main goals of the study. The spacing (x), diameter (d), and distance (y) between the center of the groove and the cavity surface was preferred. The optimal operating condition parameter of the x-value was finally obtained, and the method can be widely used in the design of conformal waterways. In 2017, Hazwan MHM [20] simulated a slot-milled square CCC using a DOE full factorial design. After the application of regression models and ANOVA methods, the cooling time obtained was the factor that had the greatest impact on warp thickness. The results showed that the response-surface method was close to the improvement rate of the straight drilling channel and the CCC design in this study.

In other words, the response-surface method can be used as the preferred method for improving and comparing research in both conventional and form-keeping cooling channels. In addition, the results of the optimization of the straight drilling channel and square milling slot channel using the response-surface method and a genetic algorithm were compared. For the best warping thickness, optimization via genetic algorithm presented a slightly different optimization point to the response-surface method; therefore, it can be seen that both the response-surface method and the genetic algorithm can give similar results to each other. From the brief review above, it is found that the best process parameters can effectively reduce warping. Few papers focus on pressure curves, which have found that variable packaging pressure curves can also effectively reduce warping. However, only the effectiveness of the packing pressure distribution is discussed. If injection pressure distribution is considered, warping will be reduced more effectively. Therefore, it is important and attractive to consider pressure distribution during the filling phase during powder-injection forming.

Short shots of melt plastic that are not fully filled into the cavity are serious and fatal defects in PIM. Small injection pressure and lower mold temperatures can sometimes lead to short shots. To avoid short shots, the easiest way is to use high injection pressure and high mold temperature. Another way to avoid short shots is to use a high melting temperature.

In 2017, Bikas [21] found the optimal gate thickness to minimize differences in filling time, combining neural networks with genetic algorithms (GA) to consider design variables in terms of mold temperature, melt temperature, injection time, and injection pressure. They approached the maximum shear stress by using a multilayer neural network, where warpage, volumetric shrinkage, and sink marks were considered objective functions. They used second-order polynomial response surfaces to approximate warpage and closure described in references. In 2018, Satoshi Kitayama [22] and others used a multi-objective optimization design for minimizing the soldering temperature by using the minimum clamping force, and the Pareto boundary between them was determined. They used the SAO of the radial-basis-function (RBF) network to identify Pareto boundaries. Numerical simulations clarified the equilibrium between the minimum welding temperature and the clamping force. In 2019, Hongwen He [23] proposed an accelerated-pedal single-foot regenerative braking control strategy based on an adaptive fuzzy control algorithm to effectively recover braking energy. Under the condition of secondary braking, a neural network controller of a composite braking system was proposed. The experimental results showed that the energy economy of IBS in three cycles was 3.67% higher than that of EU260, the braking severity was reduced, and the hydraulic braking time was 2.27% lower than that of EU260.

In 2019, Chen-Yuan Chung [24] combined finite element analysis with gradient-based algorithms and robust genetic algorithms to determine the optimal layout of cooling channels. The use of conformal cooling channels reduces surface temperature differences, spray times and warpage of the melt. The fringe pattern is improved by the optimal values of melt temperature, mold temperature, filling time and package time obtained by experimental design, eliminating local variations of birefringence.

In 2020, Chang [25] proposed a screw life prediction method based on a hybrid composite screw process-parameter method for dynamic iterative work. A combination of an automated virtual metering (VM) system and a recognizable performance evaluation (RPE) scheme was proposed. The injection of composite screws was predicted by extracting the characteristics of a given cutting condition and related process parameters from the sensor data. In 2020, Chang [26] proposed an optimization method based on the reverse warpage model; this reduces the thermal conductivity of the plastic material and the curing layer on the mold surface, which means that the shrinkage of the melting zone in the component will continue to lead to warpage. The sensitivity of warpage prediction to the relationship between the two most important material properties (glass fiber and holding time) was analyzed. In 2020, Junren Shi [27] combined the driving data of experienced drivers in different driving environments with deep learning to build a deep long-term–short-term memory network to predict the aperture of the brake pedal under different braking types. The proposed anthropomorphic control method, which combines driving data with deep learning, can be used to predict the aperture value of the pedal of the loading mechanism in a complex driving environment. The 2021 M.I.M. Sargini [28] study explored the possibility of rapidly producing brake pedals for metal cars using low-cost additive manufacturing techniques, using metallic polymer fiber filaments, followed by a debonding and sintering process. Finite Element Analysis was applied to analyze the feasibility of a new brake pedal designed for AM machining. Physical tests were conducted on metal brake pedals produced by FDM, the results of the finite element analysis were verified, and the reliability of metal-based technology was evaluated.

In 2021, Sai Li, Xi Ying Fan et al. [29] designed orthogonal experiments based on simulation results while selecting important factors affecting warpage. The kriging model was established by using four related functions that affected the accuracy of the model, and the model was optimized using a numerical optimization algorithm, a direct search method and a global search method to obtain the best injection-molding parameters that produced the minimum warpage. In 2021, Shaochuan Feng et al. [30] conducted a systematic evaluation of the design, manufacture and application of CC channels. They calculated and selected some key design parameters for CC channels related to channel shape, size, and position, taking into account cooling performance, mechanical strength, and cooling hydraulic drops. By using CC channels for uniform and fast cooling, cycle times can be reduced by 70% and shape deviations can be significantly improved. In 2021, Yannik Lockner et al. [31] trained an artificial neural network based on transfer learning of induction networks to optimize the parameters of injection flow, packing time, packing pressure, cooling time, melt temperature and cavity wall temperature. Generating four sample points of the new process could train an injection-molding process model with a certainty (R2) of 0.9 or higher, compared to an 88% reduction in training data for the traditional method. In 2021, Chil-Chyuan Kuo et al. [32] proposed a differential sensitivity-fusion method (DSFM), which used a metamodeling method to construct a response predictor and calculate the target response of any sample point in the global design space. They obtained the Pareto-optimal solution, wherein the response-predicted value was used as the fitness function. Taking the front bumper of a car as an example, numerical results showed that the method had better prediction accuracy and performance.

The cooling performance of the accompanying cooling channels was tested using digital and experimental methods. The cycle time and distortion of the brake pedal plastic were considered to be cooling properties, and the process parameters were optimized. In general, short cycles can cause large upturns, while long cycle times can cause small warps. Therefore, a trade-off between cycle time and warp was observed. The authors developed multi-objective design optimizations to determine trade-offs (Pareto Frontier). This study analyzes the modal flow simulation of pedals. The PA6 contains 40 types of fiberglass; it improves the cooling tube by comparing filling time, hold time, melting temperature, maximum cooling time, cooling to maximum temperature, etc., and will be further improved in the future. In addition, it is particularly important to shorten the process time, which is also beneficial to improve the production efficiency and economic efficiency of automobile products. Therefore, the design function is mainly to optimize the design of the cooling pipe, and to shorten the entire product-molding cycle, including filling, pressure, cooling, etc. There is also a need to reduce distortion and cooling shrinkage, which can be further optimized by optimizing the design of the cooling pipes; this can then be further optimized, for example, with better cooling effects. The purpose of this paper is to test the cooling performance of the accompanying cooling channels using digital and experimental methods. The cycle time and distortion of the brake-pedal plastic are considered to be cooling properties, and the process parameters are optimized. In general, short cycles can cause large warpages, while long cycle times can lead to small warpages. Therefore, a trade-off between cycle time and warping is observed. We developed multi-objective design optimizations to determine trade-offs (Pareto Frontier). Numerical simulations are very dense, so the SAO in the radial-basis-function network developed by one of the authors was used to identify Pareto edge faces with a small amount of simulation. Based on the numerical results, the accompanying cooling channel was used for image analysis. The purpose of our study is to obtain an empirical curve that can be based on Pareto-optimal solutions, which can provide a reference for actual production and select appropriate Pareto-optimal solution points according to actual production needs. Listed below are five points detailing the main contributions:

1. The response-surface method is used to identify the optimal arrangement of the cooling waterway, according to the cooling time, warping thickness, temperature distribution and thermal stress, and other different targets, to display the performance output of CCCs. The obtained output is evaluated.
2. Because the relationship between target and constraint is not clear, the approximate global minimum value is found by means of computationally intensive numerical simulation and by using the method of response surface (called metamodeling).
3. Multiple sampling points are set up in the design variable space. Orthogonal arrays or Latin designs (LHDs) are typically used to determine these sampling points. These sampling points are evaluated for targets and constraints.
4. A response surface is constructed that approximates the target and constraint. Radial-basis-function (RBF) networks are used to construct response faces. Approximate optimal values can be obtained by optimizing the response surface—both the response-surface optimization and the original design optimization problem of the order of the approximate optimization method (SAO).
5. The SAO radial-base-network deviation function is used to identify the cycle time and warping in the Pareto boundary, to find a highly accurate global optimal solution to different cooling time warping values.

## 3. Materials

### 3.1. Product Introduction

**Figure 1:**
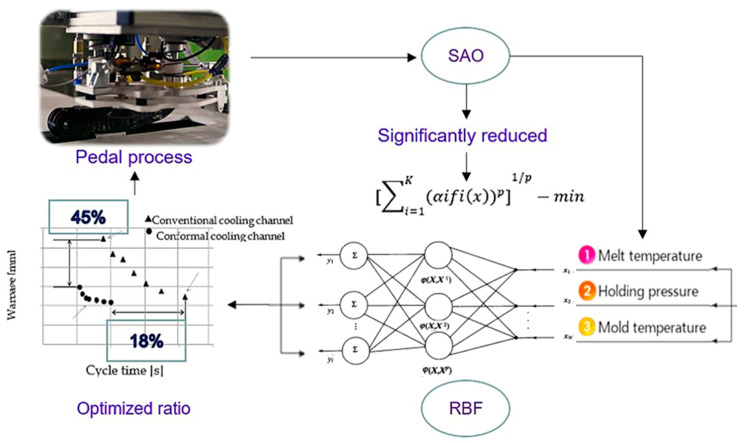
Graphical Abstract.

**Figure 2:**
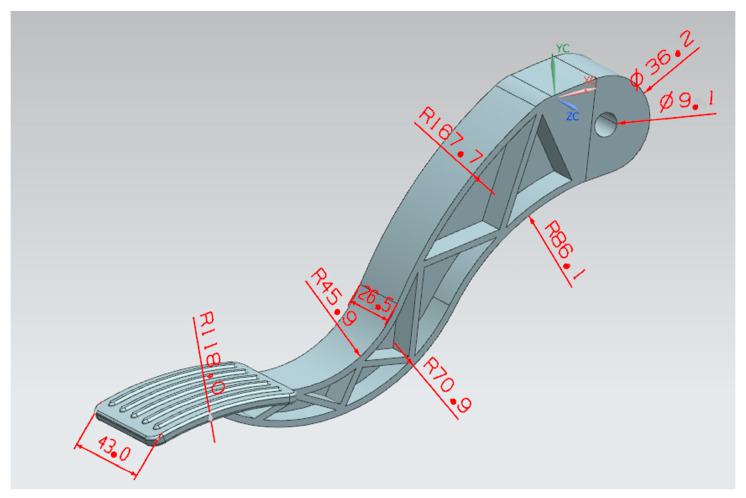
Product appearance and size.

The experimental subject was a car pedal, with pedal size as shown in Figure 1; the pedal needs to withstand a large shear force and forward impact, so we choose the material with fiberglass (PA6 AKROMIDB B3 GF 40 schwarz (3383)). The viscosity and shear curve and PVT characteristic curve are shown in Figure 2. Mold temperature and material temperature should be within the adjustment range; temperatures that are too high or too low can cause plastic cracking or the formation of defects. Because the meat thickness was larger, it was more difficult to fill the completed part, and easy for heat accumulation and sealing to occur, so the inlet selection in the connection hole was directly above. To meet the principle of filling the meat thickness as much as possible, to avoid short shot, a needle-point pouring port is generally used in a mold three-plate mode cold-flow channel system. This can be used for multi-mode dispensing or multi-fill dispensing of water. This product is suitable for small or thin-walled ejection sections (meat thickness does not apply), and needle-point pouring directly through the appearance of the ejection section. When the mold is open, the pin-point pouring port can be removed directly from the ejection section. To avoid visible pouring marks on the ejection surface, the aperture of the needle must be small enough. The recommended diameter is about 40% to 80% of the size of the meat, and the pouring length is about 0.5 to 1 mm. The default door sizes before the improvement were D-3 and 9, with the maximum meat thickness of 9.50% of the length being the large end-door diameter, with a length of 10 mm.

Because the temperature control of the mold has a great influence on the production efficiency and the quality of the plastic parts, the diameter of the cooling pores and the length of the cooling circuit are important factors in the design of the cooling circuit of the cooling system. These factors can change the heat transfer area and the flow of coolant, thereby changing the cooling effect.

The parameter values set by the mode-flow analysis software include fill time, holding pressure, hold time, plastic temperature, and waterway temperature. As shown on Table 1, the average plastic temperature was 280 degrees C. Its strength was protected by using multiple segments. In order to ensure that the product-molding effect, on the basis of optimization, would have a better effect later, the multi-segment pressure intervals were kept as uniform as possible, in order to obtain the optimal solution of Pareto.

Unlike metals, plastics have a high thermal capacity, and crystalline plastics have a higher thermal capacity than non-crystalline plastics. For example, plastics have a higher coefficient of thermal expansion than metals. One way to modify these values is to use mineral fillers such as fiberglass. As in most manufacturing areas, production time and cost (advance and lag) are closely related. The longer it takes for parts to be produced, the higher the cost, and in injection-molding production, cooling time is often considered an indicator of cycle time. Improving the cooling system can reduce production costs. An easy way to control temperature and heat exchange is to create several channels inside the mold where coolant is forced to circulate. Traditional processes, such as CNC drilling, can be used to create straight-line channels. Here, the main problem is that this is not possible in 3D, especially close to the mold wall of the complex channel. This results in an inefficient cooling system that causes warping and cooling time to increase due to the inability to remove heat-uniform molds and different shrinkages. On the other hand, if the cooling channel can conform to as many shapes as possible, then the cycle time of the cooling system can be significantly reduced, and the cooling occurs evenly in all areas.

**Figure 3:**
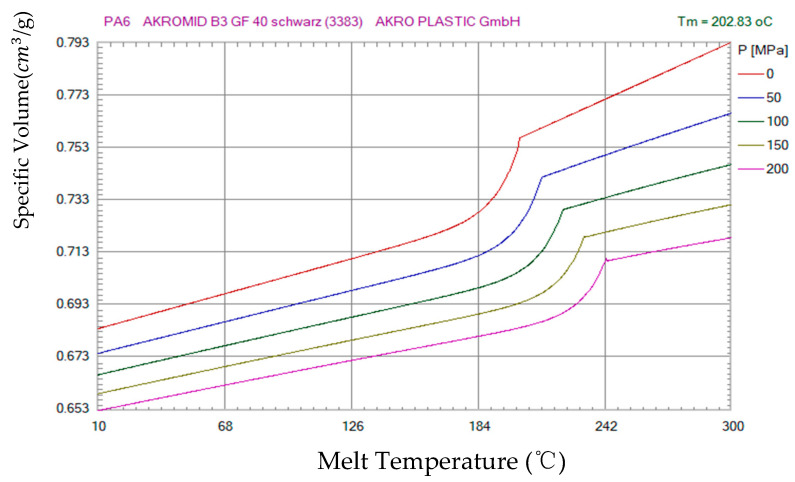
PVT diagram.

Furthermore, if the part’s temperature is the same at each point, then the subsequent contraction outside the mold is uniform, which avoids warping after the part is injected. Another advantage is that molds with pro-shaped channels reach operating temperature faster than normal molds equipped with standard (or drilled) cooling channels. This reduces the time it takes for the molding machine to start. When the polymer is injected, it immediately solidifies and touches the wall of the mold. If the part is large enough and the thickness is too small, the polymer solidification will hinder the flow and prevent the cavity from filling completely. In this case, the mold must be heated to a specific temperature to allow the polymer to flow. Despite these advantages, we can note that the new technologies involved in mold production with bonded channels increase the additional complexity of the construction process, thereby increasing the initial cost. The effectiveness of the synth channel was studied by constructing three different molds with and without co-cooling. It was shown that the latter technique brought about significant improvements and generally reduced the cycle time, while improving heat transfer. This contributes to understanding the importance of co-channels and the use of new highly conductive materials. The study shows that using nickel/copper molds with co-channels (copper layers) increases productivity by about 70% compared to conventional steel molds with drilled cooling channels. The co-shaped channel and the drilled cooling channel were compared. The research on these was based on modeling cores and cavities, and using software from both technologies, we continued to build molds to compare theoretical and experimental data. Subsequent analysis shows that the formed channel mold reaches the operating temperature faster than the conventional channel mold, obtains a more uniform temperature distribution, and has more effective heat transfer capability. Generally speaking, the cooling time is to ensure that the cooling part reaches about 90%; however, because the internal cooling time is too long, the cooling time will be greatly increased and is very small. The cooling time for traditional cooling waterways is 26 s. It is clear from Figure 3 that the shrinkage rate of the pedal part with large volume shrinkage decreases significantly, which is conducive to ensuring the size of the important face and reducing the internal maximum volume shrinkage at the connection head.

### 3.2. Injection-Molding Parameter Selection

The area of the final cooling cycle that is not cured occurs at the connection head with the largest thickness of the meat, as shown in Figure 3, close to the connection hole inside the plastic. Cooling water is not easy to achieve, and heat is not easy to dissipate. This also requires a discussion of how to build a pathway to achieve better cooling and more efficient cooling times. Time is determined by the mold’s ability to carry heat from molten polymers, shown as Table 2. The liquid passes through the cooling channel in the mold at the desired temperature. This must allow the melted polymer to flow into various parts of the cavity while removing heat as quickly as possible. Thus far, these channels have been drilled out, only in straight lines. A more efficient cooling method can be achieved if the water passages can conform to the shapes of the parts and change their cross-sections to increase the thermal conductivity area.

This also helps to reduce the warp of the part when ejected, as the plastic cools more evenly. Temperature control requires control of the temperature of the molten polymer, mold, encirclement temperature and clamping system. When molten plastic is injected into the mold, it must be solidified to form an object. Mold temperature is regulated through the circulation of a liquid cooler, which is usually water or oil flowing through the passageway inside the mold. When the part is cool enough, it can eject. Most (95%) of the contractions occur in the mold and are compensated by incoming materials; the remaining contraction occurs sometime after the part is produced. It can be seen that the warping positions that need to be optimized are mainly concentrated in the front foot position of the pedal and the large head position of the tail connection hole of the pedal, which means the distribution area of the pass-through waterway needs to be distributed as much as possible in these positions. As the temperature control of the mold has a great influence on productivity and the quality of the plastic parts, the diameter of the cooling pores and the length of the cooling circuit are important factors in the cooling design of the original process. The main reason for the shrinkage warping deformation of the product is an insufficient cooling-water setting and internal heat accumulation phenomenon, which gives the product inward contraction, with total warping of 3.85 mm.

### 3.3. Optimization of Injection-Molding Parameters

**Figure 4:**
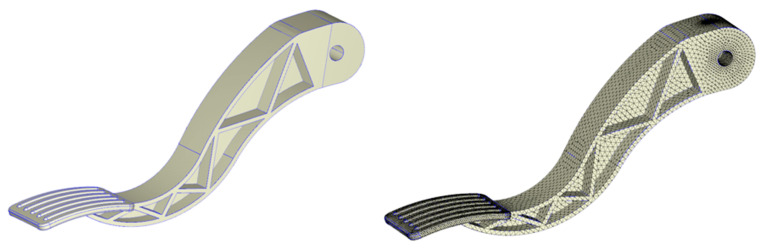
Type of meshing figure.

The optimization scheme was used to improve total warping and volume shrinkage. Using the following waterway in Figure 4, the cooling effect is better than the original process, and the plastic temperature is significantly reduced and improves the heat product phenomenon. The maximum total warping and the original process improve by 0.25 mm, by improving the pouring port. We increased the flow rate of the plastic in the filling period by 0.25 s, the inlet pressure by 0.9, and the maximum mode-locking force by 40%; this saved the station cost and time, reduced the time for cooling from top temperature by 10 s compared with the original process, and shortened the cycle time. Changing the melt temperature in the improved process leads the conclusion that a low temperature can save cooling time. Moreover, the cooling time of melt glue temperature was 22 s, 40% lower than the original process which was 35 s; the maximum warping volume was at its lowest at 0.06 mm, a decrease of 0.37%; and the filling time was 0.01 s. However, the lower the temperature of melting glue required by the maximum mode-locking force, the higher the loss of the platform.

**Figure 5:**
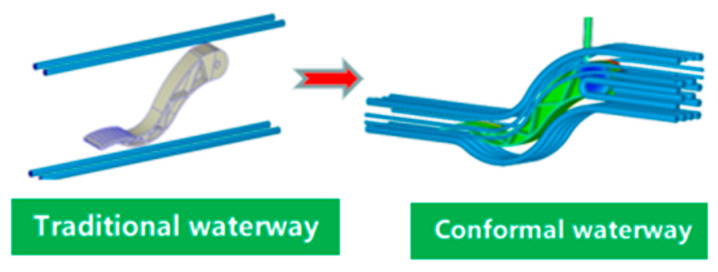
Comparison of the optimized conformal waterway and the traditional waterway.

According to the three pressure-keeping project results in Table 3, multi-holding ratio—project II is 90%–75%–60%; the volume shrinkage rate is 12.8%; and the maximum warping volume is 3.36 mm, 0.2 mm higher than the original shape; however, the maximum locking force is increased by 7.35 Mpa, so maintaining pressure will also increase the station loss. The results show that maximum warping occurs at the connection head and pedal; the original maximum warping is 0.73; maximum warping is about 0.27 after improvement; and the maximum improvement of total warping displacement is 0.46 mm. We can see that warping of the connection head part and the front of the pedal significantly improved, and the red area is shifted to an unimportant position, which is of great help to the stability and reliability of the part, as shown in Figure 5.

## 4. Methods

### 4.1. Sequence-Approximation Optimization (SAO)

In general, a multi-objective design optimization is formulated as follows:

(1) (f1(x),f2(x),…,fK(x))−min}

x∈X where *f*i (*x*) is the ith objective function to be minimized and *K* represents the number of objective functions. *x* = (*x*1, *x*2, …, *xn*). The design variables with *n* dimensions and *X* feasible regions are represented by T.

The process parameters in the PIM play a major role in changing the injection cycle time and warping. The injection-molding variable factors designed in this paper are: melt temperature (Tmelt), filling time (*fi*), pressure-holding pressure (P), and cooling time (*tc*). The lower and upper limits of the injection-molding parameter factor are shown in Table 3.

The first objective function *f*1 (*x*) is defined as period time, usually in the explicit form of the injection-molding parameter factor, along with cycle time and warping as functions to obey, as shown in Equation (2):

(2) f1(x)=tinj+tp+tc−min

The change in the warping value is defined as the second objective function, *f*2 (*x*). The results obtained through numerical simulation in Figure 5 can obviously find the change in the warping value of various parts, with the maximum warping value reached along with the direction of the right arrow.

**Figure 6:**
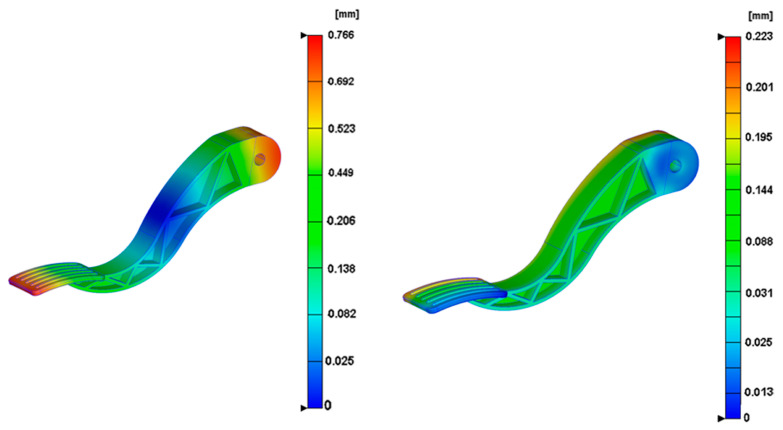
Warpage comparison.

In this paper, by defining two different objective functions as period time and warping values, the purpose is to identify the Pareto boundary point by analyzing the above two objective functions. Because the object PIM simulation studied in this paper is very dense, the identified Pareto boundary points can be considered effective. The general procedure of the summarized Sao method for identifying Pareto boundaries is shown in Flow Figure 6. The steps are described in the following text:

(Part 1) First, the initial value of the sampling point is determined, and the initial sampling point is determined by using the Latin hypercube design (LHD) method.
(Part 2) A simulated numerical analysis of the sampling points is performed, and then they are substituted into the target function for the corresponding numerical calculation.
(Part 3) Because the objective function can be analyzed and calculated through an approximate RBF network, here, the approximated objective function is marked as ~*fi* (*x*) (*i* = 1, 2, …, *K*).
(Part 4) A response surface is found by using the weighted lp norm method, and the Pareto boundary point is obtained by using the response-surface analysis. The formula is as follows:
  (3) $${\left[\sum_{i=1}^{K}{\left(\alpha ifi\left(x\right)\right)}^{p}\right]}^{1/p}-min$$

*p* is the parameter, *i* (*i* = 1, 2, *K*), and indicates the weight of the ith objective function, and the weight value is set to 4 via calculation. A set of Pareto-optimal solutions is obtained by calculating the weights assigned to each design factor. For a Pareto-optimal solution obtained via substitution Equation (3), the calculation result can be a new sampling point to update the response surface, but the algorithm can only achieve local accuracy.

(Part 5) We use the density function introduced later in this paper to find the area where the sampling point is not covered. This minimizes the function by constructing the density function first. The optimal solution obtained from the density function is then rearranged to serve as a new sampling point value. We repeat the procedure described above to terminate this procedure with the final criterion. The purpose of introducing this step is to distribute the sampling points as evenly as possible, thus laying the foundation for the global approximation.
(Part 6) If the calculation results meet the terminal criteria, the Sao algorithm will not continue to run. Instead, we return from this step to step 2.

The determination of the terminal criterion is as follows: the average error between the response-surface value of the Pareto boundary point calculated in part 4 and the numerical simulation of part 2. In order to ensure the accuracy of the average error, the Sao program is set within 5%.

**Figure 7:**
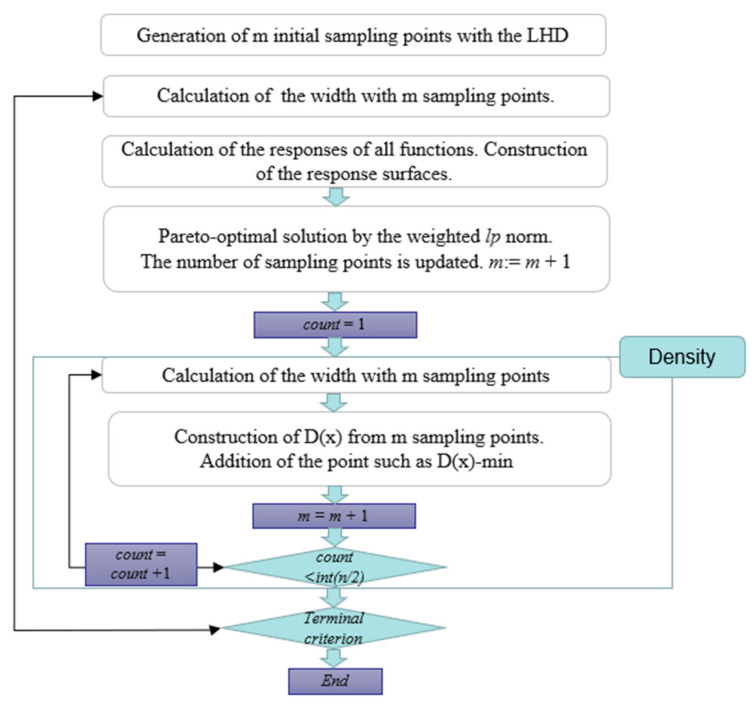
Flow of sequential approximate optimization for multi-objection.

Figure 6 shows the SAO process for multi-objective optimization issues. In this paper, the entire SAO process uses the RBF network, and this paper uses the optimal error between the response surface and the numerical simulation as the final criterion of the SAO algorithm. First, the initial sampling point is generated using the Latin Hypercube Design (LHD). Assuming that the number of sampling points is m, the optimal solution of the response surface is obtained by constructing the response surface from the m sampling point by using the radial-basis-function network. The error of the target function at the optimal solution is investigated. If the error is small, the SAO algorithm is terminated. Otherwise, the optimal solution of the response surface acts directly as a new sampling point, and the algorithm moves on to the next stage of looking for unexplored areas. At this stage, the number of sampling points is updated to m-m1. The goal of this phase is to find globally approximate unexplored areas. At this stage, the density function is constructed by a network of radial basis functions. The optimal solution of the density function is the new sampling point, and the sample points are updated as shown in Figure 7, which introduces parameter counts. This parameter controls the number of sample points that the density function can obtain. Therefore, in the proposed algorithm, the number of sampling points for density functions varies according to the number of design variables. If the parameter count is less than int (*n*/2), the parameter is increased to count = count1. The final criteria for this phase are given by int (*n*/2), where represents rounding. If the endpoint criteria for this phase are met, we will perform a numerical simulation at the new sampling point.

### 4.2. RBF Function Network

The RBF network is a three-layer feed-forward network. The output of the network *ŷ*(*x*) corresponds to the response surface, where m denotes the number of sampling points, *hj*(*x*) is the *j*th basis function, and *wj* denotes the weight of the *j*th basis function. The following Gaussian kernel is generally used as the basis function:

(4) $$hj\left(x\right)=\exp\left(-\frac{{\left(x-xj\right)}^{T}\left(x-xj\right)}{r^{2}j}\right)$$

In Equation (4), *xj* represents the *j*th sampling point, and *rj* is the width of the *j*th basis function. The response *yj* is calculated at the sampling point *xj*. The learning of RBF network is usually accomplished by solving

(5) $$E=\sum_{j=1}^{m}{\left(yj-y\left(xj\right)\right)}^{2}+\sum_{j=1}^{m}\lambda jw^{2}j-min$$

The introduction of the second term in the formula is mainly because of the regularization requirement of Equation (5). Additionally, the value of *j* (5) in the equation is usually as small as possible (for example, *j* = 1.0102). It infers that the learning ability of the RBF network is calculated to determine the weight vector w and the weight value. The results of the formula are as follows:

(6) $$r=\frac{d_{max}}{\sqrt[{n}]{nm}}$$

where *d_max_* represents the maximum distance between sampling points; *n* represents the number of design variables; and *m* represents the number of sampling points. Equation (6) applies to all base functions. Therefore, *r*1 = *r*2 = *rm* = *r*. Assume that the proportions of all design variables are equal. This scaling technique is called adaptive scaling technology, and we consider the *K*-level full-factor design, where the regular interval is ∆*d* in this case, and the *d_max_* data are determined by:

(7) $$d_{max}=\sqrt{n}\left(K-1\right)\Delta d$$

In the case of n design variables, the number of sampling points m is simply calculated as follows:

(8) $$m=K^{n}$$

We solve (8) about *r*/∆*d*, and then, we can finally obtain the following equation:

(9) $$\frac{r}{\Delta d}=n^{\frac{n-2}{2n}}\left(1-\frac{1}{K}\right)$$

It is clear from Equation (10) that the learning of the RBF network is equivalent to the matrix inversion (HTH + Λ) − 1. The new sampling points are added through the SAO process. The following simple estimate is adopted to determine the width in Equation (10):

(10) $$rj=\frac{d_{j,max}}{\sqrt{n}\sqrt[{n}]{m-1}}j=1,2,\ldots ,m,$$

where *m* is the number of sampling points, n is the number of injection-molding parameter factors, and *dj* is the distance between the *j*th sampling point and the one furthest from it.

The literature shows that Equation (10) can be successfully applied to the least-square-support rectangle machine.

In Sao’s method process, it is necessary to find a method to find the area where the sampling point is uncovered. In order to find the unexplored area using the RBF network, the function prediction can be implemented via Kriging’s method, so as to explore the area where the sampling point is uncovered using the density function. On how to construct an appropriate density function and effectively obtain the local optimal solution to the region of the sampling point:

## 5. Results

**Figure 8:**
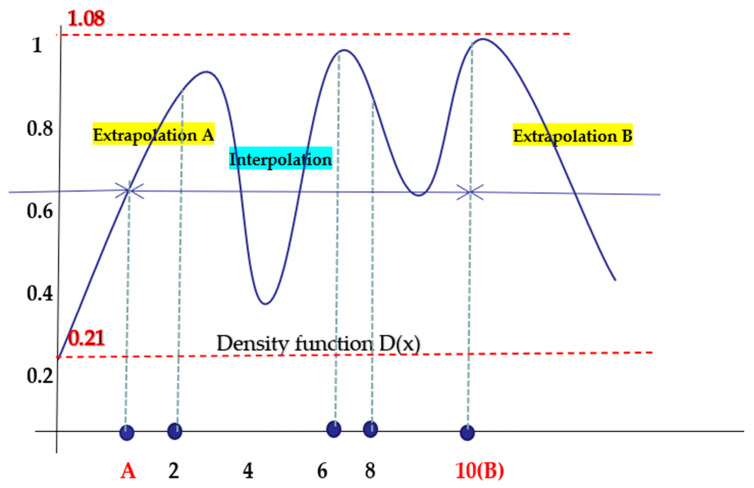
Illustrative example of density function in one dimension.

In SAO, it is important to identify unexplored areas that are globally approximated. The Kriging method can achieve this through the Expected Improvement (EI) function. In order to use the RBF network to find unexplored areas, we developed a function called density function. The process of building density functions is summarized below:

(D-step 1) The following vector *y^D^* is prepared at the sampling points.
(D-step 2) The weight vector *w^D^* of the density function
  (11) $$y^{D}={\left(1,1,\ldots ,1\right)}_{m\times 1}^{T}$$
  *D*(*x*) is calculated as follows:
  (12) $$w^{D}={\left(H^{T}H+\Lambda \right)}^{-1}H^{T}y^{D}$$
(D-step 3) The density function *D*(*x*) is minimized.
  (13) $$D\left(x\right)=\sum_{j=1}^{m}w_{j}^{D}h_{j}\left(x\right)-min$$
(D-Step 4) Figure 8 is an experimentally obtained one-dimensional Pareto-optimal solution map, or a Pareto boundary point map. It can be seen that the RBF network is basically the interpolation between the sampling points, and the black points indicate the sampling points. The local minimum is obtained in the region not involved by the sampling point, so the P1 and P2 two points in Figure 8 are the maximum and minimum of the injection-molding parameter variables of the density function. As shown in Figure 8, a count calculation is added, and int () is rounded. If the count is less than int (*n*/2), the number will rise as count = +1. This parameter limits the range of the number of new sampling points obtained via the density function. The density of the function is constantly updated and optimized to ensure global approximation as well as homogenization of the sampling point.

In this experiment, 15 initial sampling points were extracted using the LHD sampling method, and then the injection parameter value of the sampling points was replaced into the RBF network for training. We identified the Pareto boundary between the injection time and warping shown in the Figure 6 and the Sao flow chart shown in Figure 8. The blue rectangular point represents the Pareto boundary point of the traditional cooling channel, and the red star point represents the Pareto boundary point of the optimized conformal cooling channel.

From the data points of the experimental results in Figure 8: the value of the conformal cooling channel over the boundary point of the conventional cooling waterway increases by 18.4%, and the warping is worth 44.8%. This shows that the conformal cooling channel greatly improves the position of the Pareto-optimal solution point compared to the traditional cooling channel. In order to more intuitively analyze the experimental data, we chose three times—P1, P2 and P3—whose warping time and cycle time are roughly the same, and the data points with obvious characteristics. Their numerical data are compared in Table 4.

From the resulting curve, the higher the temperature of the molten plastic, the greater the pressure and time, and it is preferable to reduce warpage. For the simulation analysis of the modal flow of the pedal, since the numerical simulations were very dense, in the developed network of radial basis functions, SAO was used to identify Pareto edge surfaces with a small number of simulations. Based on the numerical results, the image analysis was performed using the included cooling channel. The result is an empirical curve that can be used in real production, with the accompanying cooling channels being determined by the designer for the values of specific parameters, to optimize efficiency in the actual production process. Here, we obtain an empirical curve based on the Pareto-optimal solution, which provides a reference for actual production and selects the appropriate Pareto-optimal solution point according to the actual production needs. If the production accuracy requirements are higher, one can try to select the best solution point of the five-pointed star below the P1 point. The characteristics of these points are the need for a longer cooling time, which will increase certain production costs, and the warpage value obtained will be reduced accordingly. If the pro-duction accuracy requirements are low, one can try to select the best solution point of the five-pointed star above the P1 point; the characteristics of these points are that the cooling time required is shorter, the production cost can be reduced, and the warpage defect obtained will be larger.

## 6. Discussion

Comparing the results of P2 and P3 in the conventional cooling channel in Table 5, the following conclusion can be obtained: For the P2 and P3 points with high packing pressure, shorter packing time, high melt temperature, and longer cooling time and cycle time, the warping value of the pedal of the P2 point is 0.25 mm lower than that of P3, which is a very obvious decrease. At the same time, it should be considered that P2 needs a longer cooling and cycle time to reduce warping, which requires a certain trade-off between warping and cycle time; this will actually greatly increase the production cost.

Therefore, the cooling channel needs to be optimized, that is, the accompanying waterway is used to effectively reduce warping without excessive time costs

Then, by comparing the experimental injection parameter data of P1 and P2 in Table 4, the following conclusion can be reached: under the same pressure and glue temperature, the cooling time and cycle time required by P1 can be shortened by about 5–6 s compared with P2; moreover, the warping values of P1 and P2 are very similar, which shows that the accompanying cooling channel can greatly reduce the cooling time and cycle time. Combined with the above experimental results, we obtain the final conclusion that high holding pressure, short packing time and longer cooling time have a positive effect on the reduction of warping, and the accompanying cooling water can effectively reduce the time cost.

**Figure 9:**
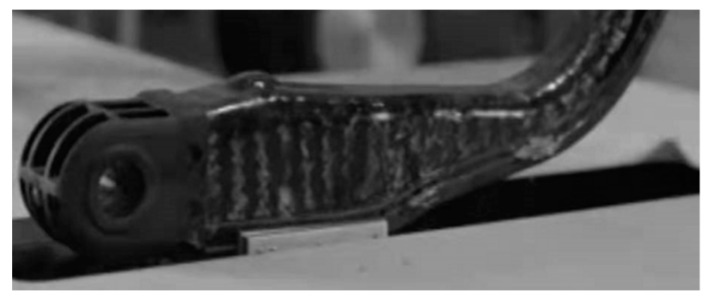
Physical pedal optimization.

Figure 9 shows the finished pedal obtained in this experiment, which uses a packing pressure of 14 Mpa, filling time of 1.8 s, melt temperature of 280 °C, cooling time of 28 s, and cycle time of 33.8 s, as shown in Table 6. The molding conditions to obtain the actual warpage are 0.35 mm; this and a warp value from the simulation analysis of the Pareto-optimal solution-point of 0.32 mm, with an error of 9.38%, is controllable; the actual required cooling time of 28 times the pedal warping defect can meet production needs. If one has higher production accuracy requirements, the cooling time and cycle time can be appropriately increased to obtain better pedal products; thus, the work of this experiment meets certain production needs, and can provide a reference for the injection-molding parameter values for the pedal parts; moreover, the conformal cooling channel of this experiment can also provide certain reference values for the actual design for pedal production.

**Figure 10:**
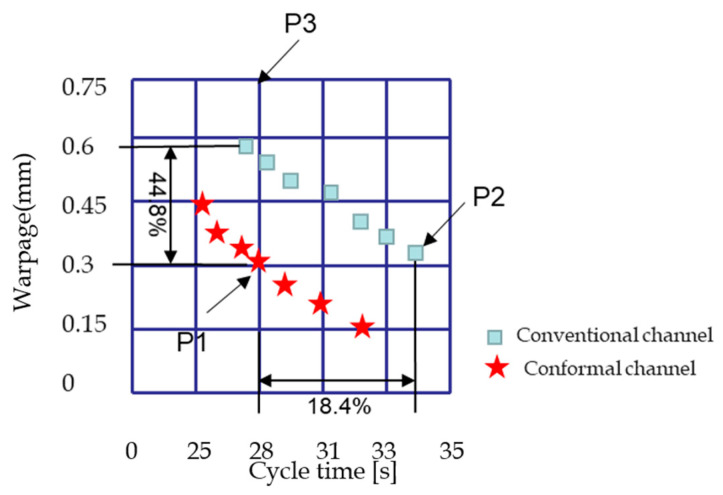
Numerical comparison of Pareto-optimal solution points between conventional and conformal cooling channels.

Therefore, shown Figure 10 in the simulation analysis of pedal mold-flow, upon putting the parameters of the experimental points into the developed radial-basis-function network, SAO was used to identify the Pareto edge surface with a small number of simulations. Image analysis was performed using the included cooling channel. The conclusion obtained is that the higher the temperature of the molten plastic during the production process, the greater the pressure and time, and the smaller the warping defect of the obtained part. The results show an empirical curve that can be used for actual production, and the accompanying cooling channel, determined by the designer, determines the value of the specific parameters to optimize the efficiency in the actual production process. The resulting empirical curve based on the Pareto-optimal solution can adjust the parameter value according to the actual production, that is, the appropriate Pareto-optimal solution point according to actual production needs. If the production accuracy requirement is higher, one can try to choose a Pareto-optimal solution point with a long cycle time; the characteristics of these points will increase the production cost, and the warping value will be reduced accordingly. If the production accuracy requirement is low, one can try to choose a Pareto-optimal solution point with a shorter cycle time; these points can shorten the injection-molding time and reduce the production cost, but the warping defects will be larger.

## 7. Conclusions

In this paper, the cooling pipe is improved by comparing filling time, hold time, melting temperature, maximum cooling time, cooling to maximum temperature, etc., and will be further improved in the future. In addition, it is particularly important to shorten the process time, which is also beneficial to improving the production efficiency and economic efficiency of automobile products. Therefore, the design function is mainly to optimize the design of the cooling pipe, and shorten the entire product-molding cycle, including filling, pressure, cooling, etc. There is also a need to reduce distortion and cooling shrinkage, which can be further optimized by optimizing the design of the cooling pipes, which can then be further optimized, for example, with better cooling effects. The cooling performance of the form-keeping cooling channel was studied numerically and experimentally. In terms of cooling performance, cycle time and warping were taken into account, and the process parameters in PIM were optimized.

In SAO, the optimal value of the response face is used as the new sampling point to improve the accuracy of local minimum values, etc. In addition, global approximation requires new sampling points in sparse areas. In order to determine sparse areas, density functions constructed by radial-basis-function networks have been developed. This density function produces local minimum values in sparse areas so that the minimum value of this function can be used as a new sampling point. In the proposed SAO algorithm, the density function is constructed repeatedly until the terminal criteria are met. As a result, many new sampling points can be obtained. Through typical mathematical and engineering optimization problems, Gaussian functions are used as base functions to check the width of Gaussian functions that affect the accuracy of the response surface. The equivalencies of the Kriging function and the real function are used as nuclear functions to identify the Pareto boundary between cycle time and warping using the deviation function of the radial-basis-function network.

We can refer to the seven initial sampling points and use the RBF network to identify the Pareto front between cycle time and warping via SAO. It can be concluded that the Pareto front of the cooling channel was greatly improved compared with the traditional cooling channel. The cycle time was increased by up to 18.4%, and the warping value was reduced by 44.8%. Numeric data are listed in the table. High pressure, long hold time, low melt temperature, high cooling temperature, and cold time are effective in reducing the curl. The cycle then the cold channel effectively reduce warping deformation. In addition, high-precision optimal solutions and response surfaces can be obtained. From the resulting curve, a larger plastic-melting temperature and a larger pressure and time are preferred to reduce warping. For modal flow simulation analysis of the pedal, because numerical simulations are very dense, SAO in the developed radial-basis-function network was used to identify Pareto edge faces with a small number of simulations. Based on the numerical results, the accompanying cooling channel was used for image analysis. The result is an experience curve that can be used for actual production, with the accompanying cooling channel being optimized for efficiency in the actual production process by the designer determining the value of specific parameters. The purpose of our study is to obtain an empirical curve that can be based on Pareto-optimal solutions, which can provide a reference for actual production and select appropriate Pareto-optimal solution points according to actual production needs.

## Figures and Tables

**Table 1:** Properties of PA6 AKROMID B3 GF 40 schwarz (3383) MECH material (data source: Moldex3D material library).

Properties	Detail
Commercial product name	PA6
Thermal expansivity (1/K)	7.41 × 10^−5^
Recommended mold temperature (°C)	80
Recommended melt temperature (°C)	280
Fiber content (%)	40
Ejection temperature (°C)	90
Modulus of elasticity (MPa)	52,600
Poisson ratio	0.32
Shear modulus (MPa)	19,100

**Table 2:** Range of injection-molding parameters.

Improvements to Plastic Temperature	260 °C	270 °C	280 °C
Cooling time required (s)	22	23	25.8
Maximum temperature	144 °C	148 °C	151 °C
Maximum warping volume (mm)	3.578	3.647	3.639
Fill-in time (s)	1.876	1.837	1.866
Volume shrinkage rate (%)	13.1	13.6	13.47
Maximum mode-locking force (Mpa)	13.7	10.6	8.7

**Table 3:** Injection-molding parameter selection table.

Set Multiple Pressure-Protection Methods with Different Pressures	Time (s)	Multi-Holding Ratio—Project I (%)	Multi-Holding Ratio— Project II (%)	Multi-Holding Ratio—Project III (%)
Time (s)	0~4.15	100	90	60
	4.15~5.53	90	75	50
	5.53~6.92	80	60	40
Volume shrinkage rate	Unit of (%)	12.9	12.8	13.1
Maximum warping volume	Unit (mm)	0.78	0.58	0.36
Average warp volume	Unit (mm)	1.86	1.88	2.02
Maximum mode-locking force	Unit (Mpa)	23.86	21.05	13.7

**Table 4:** Range of research factors.

Tmelt (°C)	*fi* (s)	*P* (Mpa)	*tc* (s)	Range
260	1.8	10	5	≧
280	2.2	14	22	≦

**Table 5:** P1, P2 and P3 point injection-molding parameter results.

	Packing Pressure [Mpa]	Filling Time [s]	Melt Temperature	Cooling Time [s]	Cycle Time [s]	Warpage [mm]
P1 (conformal channel)	14	1.8	280	28	33.8	0.32
P2 (conventional channel)	14	1.8	280	33.5	39.3	0.33
P3 (conventional channel)	10	2.2	260	26.8	33.2	0.58
Improvement rate				25.5%	18.4%	44.8%

**Table 6:** Specification of optimal parameter values.

Optimal Solution Parameters	Packing Pressure [Mpa]	Filling Time [s]	Melt Temperature	Cooling Time [s]	Cycle Time [s]	Warpage [mm]
	14	1.8	280	28	33.8	0.32

## Data Availability

Not applicable.
